# A Dutch Nationwide Bariatric Quality Registry: DATO

**DOI:** 10.1007/s11695-017-3062-2

**Published:** 2017-12-22

**Authors:** Youri Q.M. Poelemeijer, Ronald S.L. Liem, Simon W. Nienhuijs

**Affiliations:** 1 Dutch Institute for Clinical Auditing, Scientific Bureau, Leiden, Netherlands; 2 Department of Surgery, Leiden University Medical Center, Albinusdreef 2, 2333 ZA Leiden, Netherlands; 3 Department of Surgery, Groene Hart Hospital, Gouda, Netherlands; 4 Department of Surgery, Catharina Hospital, Eindhoven, Netherlands

**Keywords:** Clinical auditing, Registry, Nationwide, Bariatric surgery, Netherlands, DATO, Dutch Audit for Treatment of Obesity, DICA, Dutch Institute for Clinical Auditing, Obesity

## Abstract

**Introduction:**

In the Netherlands, the number of bariatric procedures increased exponentially in the 90s. To ensure and improve the quality of bariatric surgery, the nationwide Dutch Audit for Treatment of Obesity (DATO) was established in 2014. The audit was coordinated by the Dutch Institute for Clinical Auditing (DICA). This article provides a review of the aforementioned process in establishing a nationwide registry in the Netherlands.

**Materials and Methods:**

In collaboration with the DATO’s scientific committee and other stakeholders, an annual list of several external quality indicators was formulated. This list consists of volume, process, and outcome indicators.

In addition to the annual external indicators, the database permits individual hospitals to analyze their own data. The dashboard provides several standardized reports and detailed quality indicators, which are updated on a weekly base.

**Results:**

Since the start, all 18 Dutch bariatric centers participated in the nationwide audit. A total of 21,941 cases were registered between 2015 and 2016. By 2016, the required variables were registered in 94.3% of all cases. A severe complicated course was seen in 2.87%, and mortality in 0.05% in 2016. The first-year follow-up shows a > 20% TWL in 86.1% of the registered cases.

**Discussion:**

The DATO has become rapidly a mature registry. The well-organized structure of the national audit institution DICA and governmental funding were essential. However, most important were the bariatric teams themselves. The authors believe reporting the results from the registry has already contributed to more knowledge and acceptance by other health care providers.

## Introduction

Bariatric surgery has already been proven as the only long-term effective treatment option for morbid obesity in terms of weight loss and comorbidities reduction [1–4]. Although this effect is nowadays embedded in several guidelines and accepted by most practitioners, still some resistance exists [5, 6]. Especially for bariatric surgery, showing outcome transparently by clinical auditing is of utmost importance [7]. This should not only consist of the clinical outcomes, but also process indicators and patient-reported outcomes should be included as well [8, 9]. For this purpose, a registry was necessary for structured evaluation of bariatric surgical care.

### History

In the Netherlands, the number of bariatric procedures increased exponentially in the 90s [10]. To deal with this increase, various health insurers started to keep track of their own individual quality indicators. The result was a fragmented and incomparable list of outcomes between various healthcare providers.

In order to define comparable outcomes, healthcare professionals took the initiative themselves. In 1996, the bariatric institutions of Belgium, the Netherlands, and Luxembourg united into the BeNeLux Association of Bariatric Surgeons (BABS). This was an improvement for scientific research. However, for the improvement of quality in healthcare, the differences between countries seemed to be a burden.

This led to the formation of a national working group for bariatric surgeons in the Netherlands, initiated by the Dutch Society for Gastrointestinal Surgery (DSGS), which was a subsidiary association of the Association of Surgeons of the Netherlands (ASN). This working group continued in April 2011 as the Dutch Society for Metabolic and Bariatric Surgery (DSMBS) and is now also the official national chapter of the International Federation for the Surgery of Obesity and Metabolic Disorders (IFSO).

### Registries

At the end of the 90s, only a few local initiatives were launched echoing various European registries. A commonly used system in the early 2000’s was the Patients Outcome Measurement Tool (POMT), originally co-funded by a medical device supplier. Some users regarded the interference of industry as a restriction, others experienced some technical drawbacks. Due to the large input of international incomparable data, the results were difficult to interpret for each individual hospital.

Most bariatric centers, not using POMT, had their own hospital ICT system or used Microsoft Excel as a database management system. Derived from POMT or homemade systems, data could be used for iBAR (international BAriatric Registry). This European registry was launched in 2008 by the European Accreditation Council for Bariatric Surgery (EAC-BS). The aim of this registry was the creation of guidelines that could be applied to different global areas and define surgeon’s credentials and institutional requirements for safe and efficient management of morbidly obese patients. The implementation of these guidelines would be applied by IFSO regional chapters in collaboration with the national bariatric and metabolic societies. In Europe, Middle East, and Africa, the IFSO European Chapter (IFSO-EC) was authorized to approve these “Centers of Excellence” (COE) in collaboration with the European Accreditation Council for Bariatric Surgery (EAC-BS).

**Fig. 1:**
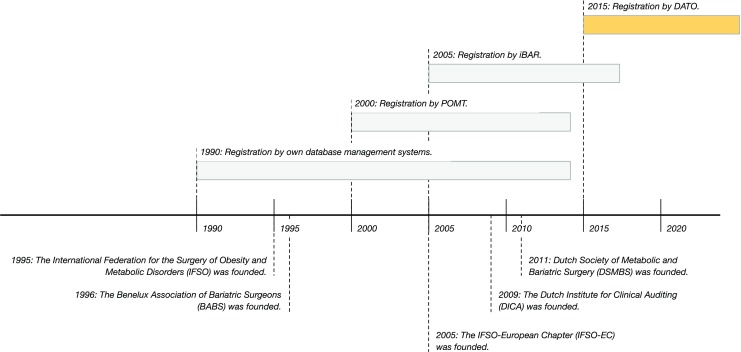
A timeline about the DATO’s origin

Despite the promising start, the international data were too difficult to interpret and comparison between countries was complicated by European laws. In addition, the mandatory set contained too many variables. Due to this large number of variables, there was an insufficient focus on the processes and outcomes of the delivered care. Therefore, this registry was not suitable for a nationwide mandatory registry (Fig. 1).

### DICA

**Fig. 2:**
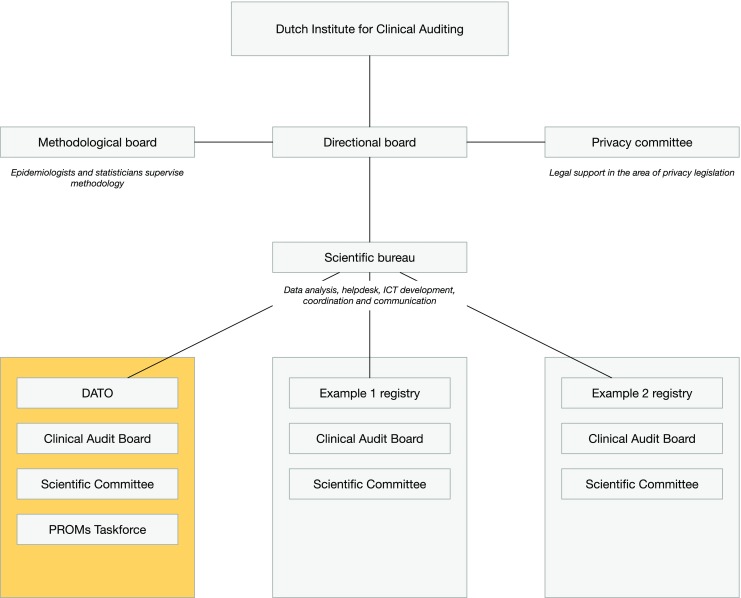
Organisational structure of the Dutch Institute for Clinical Auditing (DICA)

A successful Dutch example of clinical auditing was the Dutch Surgical Colorectal Audit (DSCA), born from the demand for national quality registries in the surgical field [11]. From this initiative, the Dutch Institute for Clinical Auditing (DICA) was founded in 2009. DICA now has 23 national registries, which facilitates clinical audits for 15 surgical and non-surgical societies. DICA consists of a directional board, management board, methodological board providing supervision of applied methodology, privacy committee providing supervision on privacy issues, and a scientific bureau facilitating a sound board for the registries (Fig. 2).

### Aim

The aim of this manuscript was to provide a review of the aforementioned process in establishing a nationwide registry in the Netherlands, with the Dutch Audit of Treatment of Obesity (DATO) as a result.

## Methods

### Funding

One of the important goals of the DSMBS was to establish a nationwide registry. In 2012, the DSMBS announced the start of a new nationwide mandatory registry. The funding arose from a special quality improvement grant from the umbrella organization of nine health insurers in the Netherlands, called “Zorgverzekeraars Nederland” (ZN). ZN offered a financial structure to establish and maintain this nationwide audit. In cooperation with DICA, the Dutch Audit for Treatment of Obesity (DATO) was established in 2014. Structural funding is currently provided by the same umbrella organization. The audit has officially started on January 1, 2015.

### Scientific Committee

A scientific committee and a clinical audit board (CAB) was put in charge of overseeing its long-term goals and monitoring the quality of the registry.

The scientific committee represents all 18 bariatric centers and all members are mandated from the practicing hospital where they are employed. As a result, all practicing hospitals have an influence on the decision making within the scientific committee. In addition, the scientific committee has the task of assessing the quality and feasibility of (international) scientific applications.

The scientific committee provides three mandated deputies for the CAB. The CAB consists of a chairman, a secretary, and a treasurer and is responsible for day-to-day running of the registry. Any decision taken by the CAB must be officially reported to the scientific committee.

### Patient Selection

The nationwide database covers all bariatric procedures in the Netherlands. The inclusion criteria for primary bariatric surgery in the Netherlands are linked to stringent requirements which are bundled in the Dutch Morbid Obesity Directive [12]. These inclusion criteria were defined by international literature and expert opinions [4, 13, 14].

Patients must be 18 years or older and must be sufficiently healthy to undergo general anesthesia and surgery. In addition, they must have a body mass index (BMI) of ≥ 40.0 kg/m^2^, or a BMI ≥ 35.0 kg/m^2^ in combination with at least one of the 6 major obese-related comorbidities: diabetes mellitus [1, 2, 15], hypertension [1, 15], dyslipidemia [15], obstructive sleep apnea syndrome (OSAS) [16], gastroesophageal reflux disease (GERD) [17], and musculoskeletal pain [18]. Weight loss as a result of intensive treatment prior to surgery (in patients who reached a weight below the minimum BMI indication for surgery) is not a contraindication for planned bariatric surgery.

Bariatric surgery is contraindicated if patients suffer from severe psychological problems, been addicted to alcohol [19], drugs [20] or other substances, an active gastrointestinal disease, or a disease that is life threatening on short terms.

### Registration

The surgical department is primarily responsible for all the data entry. Some hospitals decided to transfer the responsibility of screening and follow-up data to other institutions like the Dutch Obesity Clinics (NOK). An overview of parameters recorded in DATO was given in Table 1.

**Table 1:** Variables recorded in DATO

Section (dataset)	Variable	Baseline	Follow-up
Patient characteristics	Social security number	M	–
	Date of birth	M	–
	Sex	M	–
	Alive/dead status	M	M
Screening	Weight	M	M
	Highest weight	R	–
	Length	M	–
	Hypertension	M	M
	Diabetes mellitus	M	M
	Dyslipidemia	M	M
	GERD	M	M
	OSAS	M	M
	Musculoskeletal pain	M	M
	Charlson Comorbidity Index	R	–
Abdominal history	If yes—8 sub-items could be answered^a^	R	–
Bariatric history	If yes—5 sub-items could be answered^b^	R	–
Procedure	Date of operation	M	–
	Name/code of surgeon	R	–
	ASA score	M	–
	Type of surgical procedure	M	–
	Clavien-Dindo Classification of Surgical Complications	M	–
Follow-up	Evaluation comorbidities	–	M
	Complications during previous period^c^	–	M
PROMs	RAND-36	M	M

*ASA* American Society of Anesthesiologists, *M* mandatory, *R* recommended

^a^Surgical interventions of hernias, stomach, duodenum, liver, biliary tract, pancreas, small intestine, appendix, colon, and/or rectum by laparoscopy or laparotomy

^b^Year of operation, type of surgery, type of technique, and/or hospital

^c^As defined by Clavien-Dindo Classification of Surgical Complications

For identification of unique patients, social security number, surname, date of birth, and sex are mandatory and registered. This patient’s traceable data is anonymized by a data processing company before analyzes taken place. Therefore, all data is anonymous for people outside the hospital.

### Screening

The registration of the pre-operative comorbidities occurs when the specific condition is present on the day of screening. Comorbidity is thus given in the registry as a yes/no option. To predict the postoperative mortality, the Charlson Comorbidity Index (CCI) is registered [21, 22]. As for diabetes mellitus, hypertension, dyslipidemia, GERD, and OSAS, a few sub-items are registered like the use of medication and laboratory tests.

To chart the surgical history, 10 main surgical areas are specified: surgical interventions of hernias, stomach, duodenum, liver, biliary tract, pancreas, small intestine, appendix, colon, and rectum. In addition, a second item registers which bariatric procedure has taken place in the past.

### Procedure and Follow-up

Registration of the operation date and type of procedure with corresponding details is mandatory. A maximum of 5 procedure-specific items are requested per procedure. Complications are scored using the Clavien-Dindo Classification of Surgical Complications (CDC) [23].

The follow-up consists of postoperative weight registration, monitoring of pre-operative registered comorbidities, and any (long-term) complications (Table 1). The follow-up will be recorded at 3, 6, 9, 12, 24, 36, 48, and 60 months, depending on the hospital and the applicable protocol. Each patient must be seen at least once a year (Table 2).

**Table 2:** Annual quality indicator DATO report

Number	Indicator	2015			2016		
		*N*	*D*	%	*N*	*D*	%
	Process						
2	Percentage of complete registered patient records regarding primary and/or secondary surgery.	9534	10,355	92.1	10,922	11,586	94.3
3	Percentage of primary operated patients, meeting the inclusion criteria on the basis of BMI and age.	8371	8756	95.6	9625	10,028	96.0
4	Percentage of primary operated patients, who are lost to follow-up in the first year after primary surgery.	–	–	–	131	6433	2.04
	Outcome						
5	Percentage of primary and/or secondary operated patients, with severe complications (CDC grade 3 or higher) within 30 days after surgery.	305	10,355	2.92	332	11,586	2.87
6	Percentage of primary and/or secondary operated patients, with a postoperative intervention within 30 days after surgery.	294	10,355	2.84	316	11,586	2.73
7	Percentage of primary operated patients, with more than 50% excess weight loss (%EWL) in the first year after primary surgery.	–	–	–	5346	6433	83.1
8	Percentage of primary operated patients, with more than 20% total weight loss (%TWL) in the first year after primary surgery.	–	–	–	5538	6433	86.1

*N* numerator, *D* denominator, *CDC* Clavien-Dindo Classification of Surgical Complications, *BMI* body mass index

### RAND-36

Patient-reported outcomes (PROs) are measured with the RAND 36-item Health Survey (RAND-36). The RAND-36 has been developed within the framework of the RAND Health Science Program in the USA. The questionnaire is identical to the MOS SF-36 questionnaire, but contains another scoring algorithm. The RAND-36 measures 8 health domains: physical functioning, role limitations caused by physical health problems, and role limitations caused by emotional problems, social functioning, emotional well-being, vitality, pain, and general health perception [24–26].

### Data Entry

There are two methods to provide the required data for DATO. The first method is by a so-called batch file, where the hospital itself extracts the necessary data from its own electronic health records software. A second option uses a secure web-based registration interface, offered by DICA. The PROs are measured in a separate database and can be cross-matched with the clinical database.

### Data Quality

To increase data quality, a clear definition is set for each data entry point with an additional explanation mark. If impracticable values or the data yields outside its predefined range, an error message occurs. A second safety measure is an automatic generated alert list, with a list of all incomplete mandatory variables for each patient record.

Once every 2 years, DICA facilitates monitoring of data quality by an external organization. Trained personnel randomly verify hospital data entered in DATO with their own electronic patient records. The results of all randomly chosen hospitals are discussed and assessed by an external quality committee. The results and recommendations will eventually be presented in an online accessible report.

### Quality Indicators

In collaboration with the DATO’s scientific committee, professional societies, hospital organizations, Dutch Patient Federation (DPF), and the health insurance companies, an annual list of external quality indicators is formulated. Indicators were derived from the international literature or written on a consensus-based development process within the scientific committee. The list is annually approved and accredited by various stakeholders. In relation to quantity and quality, the minimum volume was set by DSMBS at 100 procedures per individual hospital in 2015 and 2016.

To analyze the different aspects of the surgical process, there are three types of quality indicators. The structure indicator provides information about the amount of bariatric procedures. The process indicators provide information about the completeness of registered (mandatory) variables to calculate all other indicators, correctness of the individual indication for bariatric surgery, and the lost to follow-up. The outcome indicators focus on clinical outcomes after bariatric surgery and possible surgical and non-surgical complications.

The lost to-follow-up indicator provides insight into the number of patients who are no longer visiting the outpatient clinic in their own hospital. The registration year for indicators with follow-up data runs from September to September. In these cases, there are no patients wrongly considered missing when their appointment falls within 12 to 14.5 months after the primary surgery date. This also applies to the indicator excess weight loss (EWL) and total weight loss (TWL).

Excess weight loss (EWL) is calculated using the formula $\frac{\mathrm{initial}\  \mathrm{weight}-\mathrm{postoperative}\  \mathrm{weight}}{\mathrm{initial}\  \mathrm{weight}-\alpha }$, with reference point *α* as an ideal BMI of 25 kg/m^2^. Total weight loss (TWL) is calculated with the formula $\frac{\mathrm{initial}\  \mathrm{weight}-\mathrm{postoperative}\  \mathrm{weight}}{\mathrm{initial}\  \mathrm{weight}}$ [5, 27].

### Benchmark

**Fig. 3:**
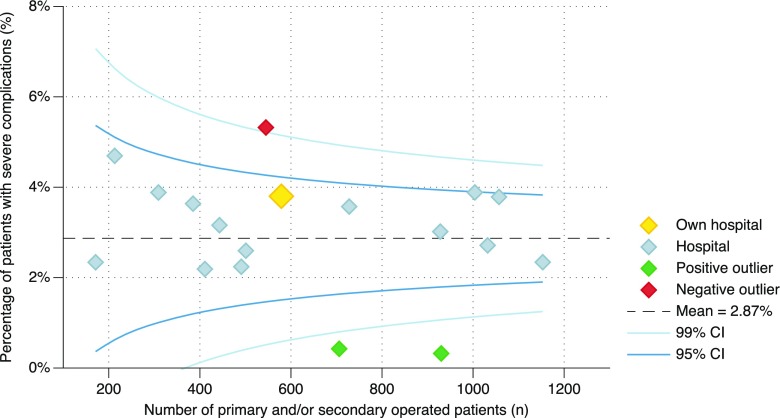
Percentage of primary operated patients in 2016, registered in the Dutch Audit for Treatment of Obesity (DATO), with severe complications (Clavien-Dindo grade 3 or higher) within 30 days after surgery, as reported per hospital

The database permits individual hospitals to analyze their own data. The dashboard provides several standardized reports and detailed quality indicators, which are updated on a weekly basis in a secured web-based environment, called myDATO. Participating hospitals recognize their own results in these funnel plots from a highlighted dot. The results of any other hospital are shown with an anonymous gray dot (Fig. 3).

### Analysis

Differences between patient and treatment characteristics were described using frequency tables. Categorical variables were compared using the chi-square trend test. Results of quality indicators and outcomes were presented concerning patients who had primary and/or secondary surgery from January 1, 2015, until December 31, 2016. Differences in quality indicator results over time were calculated with the chi-square trend test.

R version 3.4.1 is used for statistical analysis in combination with the “Companion to Applied Regression”-package (car 2.1–5) and “A Grammar of Data Manipulation”-package (dplyr 0.7.2).

## Results

Between 2015 and 2016, a total of 21,634 unique patients were registered in the DATO, with a total record count of 21,941. Of these, 18,784 (85.6%) operations were primary procedures. The mean age was 43.8 years (± 11.2 SD), with a median of 44 years. The mean BMI was 43.3 kg/m^2^ (± 5.4 SD) and median of 42.3 kg/m^2^.

The largest group of procedures involved patients with a Roux-en-Y gastric bypass (RYGB) (72.4%; *n* = 15,889), followed by gastric sleeve (GS) (17.7%; *n* = 3885), one anastomosis gastric bypass (OAGB) (5.9%; *n* = 1298) and other procedures (4.0%; *n* = 869).

### Structure Indicator

All 18 bariatric centers met the quality indicator regarding a minimum of 100 bariatric procedures per individual hospital, with a range of 171 to 1153 procedures.

### Process Indicators

The process indicator defined as completeness of the registered patient, which means all mandatory variables were registered in DATO to calculate the indicators, revealed a 92.1% (*n* = 9534) completeness in 2015, which increased in 2016 to 94.3% (*n* = 11,586).

In 2015, 95.6% (*n* = 8371) of the cases met the requirements for bariatric surgery, which increased in 2016 to 96.0% (*n* = 9625). In 0.8% (*n* = 169) of all registered cases, the BMI were unknown, 2.0% (*n* = 431) had an unknown presence of any comorbidity, and in 0.02% (*n* = 5), the age could not be calculated.

In 2016, the lost to-follow-up percentage was 2.04% (n = 131) of the 6433 primary bariatric procedures performed from January to October 2015.

### Outcome Indicators

The first measured outcome indicator was mortality, also measured as CDC grade 5, within 30 days after surgery or during the same hospital stay. In 2015, 10 patients (0.1%) died after surgery; whereas, 6 patients (0.05%) died in 2016.

The postoperative complicated course within 30 days after surgery or during the same hospital stay was measured by CDC grade 3 or higher. Grade 4 was described as life-threatening complications requiring intensive care admission, which occurred 65 times (0.6%) in 2015 and 91 times (0.8%) in 2016. Requiring surgical, endoscopic, or radiological intervention (grade 3) had to take place 230 times (2.2%) in 2015 and 235 times (2.0%) in 2016. Added together, any complication during admission occurred in 3.0% (*n* = 305) of the cases in 2015 and 2.8% (*n* = 322) in 2016.

In 2016, 83.1% (*n* = 5346) of the operated patients from January 2015 till October 2015 had reached more than 50% EWL after primary surgery. The group with the highest percentage of > 50% EWL was OAGB (86.8%; *n* = 275), followed by RYGB (85.0%; *n* = 4218), GS (72.3%; *n* = 825), and other procedures (34.5%; *n* = 29).

From January 2015 till October 2015, 86.1% (*n* = 5538) of the operated patients succeeded more than 20% Total Weight Loss (TWL) after primary surgery at the first-year follow-up in 2016. The highest percentage of >20% TWL, was measured at OAGB (90.2%; *n* = 286), followed by RYGB (87.2%; *n* = 4325), GS (78.8%; *n* = 899) and other procedures (34.5%; *n* = 29).

## Discussion

This manuscript provided an extensive and complete overview of the aforementioned process in establishing a nationwide registry in the Netherlands, with the Dutch Audit of Treatment of Obesity (DATO) as a result.

DATO was mandatory for all bariatric centers, and therefore it was required to register all bariatric procedures. This was a requirement of the insurance companies to carry out bariatric surgery. DATO provided a nationwide transparent overview and results of bariatric procedures. By identifying positive outliers based on benchmarked indicators, DATO can provide healthcare professionals with actable information to improve their care and patients with valid information to choose a hospital of their preference.

### Clinical Auditing

The cornerstone of effective auditing is to provide high quality standards for entering data in an online accessible tool, using uniform international definitions, and producing interactive feedback charts for individual healthcare centers to improve care where necessary. Only when all surgeons and healthcare centers are participating in the registry, valid conclusion can be drawn from the provided benchmark information [11, 28, 29]. In the first years of registration, DATO succeed in the mission of high quality data, national coverage, and providing useful benchmark information for the individual clinic [30].

### Complicated Course

Bariatric procedures were considered relatively safe, regarding to other surgical interventions, where mortality and morbidity were considered acceptable [1, 4, 31, 32]. With 16 deaths out of 21,634 unique patients in the past 2 years, bariatric surgery in the Netherlands can be considered relatively safe. A severe complication during admission was characterized by CDC grade 3 or higher. This occurs in 2.9% of patients. It is remarkable that in about 0.8% of cases, the “complication” involved a diagnostic laparoscopy. In bariatric surgery, however, this is considered a valuable diagnostic tool. When compared to international literature, the number of serious complications was significantly lower in DATO [4, 31].

### Limitations

The DATO dataset contains a large set of data points to cover a wide variety of bariatric treatment characteristics. This is associated with a substantial administrative burden, because bariatric surgeons are responsible for providing their own surgical and follow-up data. Nevertheless, the dataset is limited and needs careful evaluation on a yearly base to prevent adverse grow. Technological innovation will contribute to higher data quality and smoother registration processes. In addition, it will be possible to get more useful information from other sources of registration to improve patient care.

Because the data provided by hospitals is self-reported, data fraud is a possible adverse effect. Therefore, an independent third-party visits bariatric centers and produces discrepancy reports to validate the data of individual centers. Bariatric centers receive the report and use it to improve the quality of data entry by their bariatric surgeons or trained personnel. A third limitation concerns the content of the DATO. From the start, the audit aimed to work together with paramedics and post-bariatric care providers. However, there are some privacy issues, and therefore it has been decided to focus primarily on bariatric surgery for now.

### Future Perspectives

DATO was designed with the idea that registering clinical information is not sufficient to give a total view of the outcomes of the treatment of bariatric surgery. It was immediately decided by the scientific bureau to measure PROs as well. Because these two instruments could technically not directly be linked, the PROs are measured in a separate database. A cross-matching with the clinical database is planned. For further improvement, initiatives are currently being undertaken for comparison with other European registries.

## Conclusion

The Dutch Audit for Treatment of Obesity has become rapidly a mature registry. The well-organized structure of the national audit, the cooperation with DICA, and governmental funding are essential. However, most importantly were the bariatric surgeons themselves: unconditional nationwide participation including very high response for PROMs. The authors believe reporting the results from the registry has already contributed to more knowledge and acceptance by other health care providers, improved quality as each center got feedback about its performance, and improved discussion with health organizations such as insurance companies about quality and indicators. This provides enthusiasm for the future.
